# Relationship between post-traumatic amnesia and white matter integrity in traumatic brain injury using tract-based spatial statistics

**DOI:** 10.1038/s41598-021-86439-0

**Published:** 2021-03-25

**Authors:** Min Jye Cho, Sung Ho Jang

**Affiliations:** Department of Physical Medicine and Rehabilitation, College of Medicine, Yeungnam University, 317-1, Daemyungdong, Namku, Daegu, 705-717 Republic of Korea

**Keywords:** Neurological disorders, Outcomes research

## Abstract

This study used tract-based spatial statistics to examine the relationship between post-traumatic amnesia (PTA) and white matter integrity in patients with a traumatic brain injury (TBI). Forty-seven patients with TBI in the chronic stage and 47 age- and sex-matched normal control subjects were recruited to the study. Correlation coefficients were calculated to observe the relationships among the PTA duration, white matter fractional anisotropy (FA) values, and mini-mental state examination (MMSE) results in the patient group. Both before and after Benjamini–Hochberg (BH) corrections, FA values of 46 of the 48 regions of interests of the patient group were lower than those of the control group. The FA values of column and body of fornix, left crus of fornix, left uncinate fasciculus, right hippocampus part of cingulum, left medial lemniscus, right superior cerebellar peduncle, left superior cerebellar peduncle, and left posterior thalamic radiation (after BH correction: the uncinate fasciculus and right hippocampus part of cingulum) in the patient group were negatively correlated with PTA duration. PTA duration was related to the injury severity of eight neural structures, each of which is involved in the cognitive functioning of patients with TBI. Therefore, PTA duration can indicate injury severity of the above neural structures in TBI patients.

## Introduction

Traumatic brain injury (TBI) is a common neurologic disorder that is associated with disability. Post-traumatic amnesia (PTA), which is a transitory state from onset to the return of orientation and the resumption of persistent memory for events, has been used widely as a criterion for classifying injury severity in TBI (mild TBI: 0–1 day PTA; moderate TBI: > 1 and ≤ 7 days PTA; severe TBI: > 7 days PTA)^1–3^. Approximately 70% of patients with TBI experience PTA and may report the presence of confusion, agitation, and the lack of attention, self-awareness, and executive function^4^. PTA has long been considered one of the strongest predictors of global outcome in severe TBI, and it is used as an instrument for determining the required level of patient supervision, as well as the timing and planning of discharge^4,5^. In addition, PTA has been demonstrated to have more precise predictability of outcome, in terms of functional independence level, disability severity, and supervision-requiring level, than the Glasgow Coma Scale and the loss of consciousness in TBI^5–7^. As a result, PTA has been reported to be a strong predictor of long-term functional outcome, return to employment, and cognitive impairment in TBI^5–7^. Therefore, elucidation of the neural structures that are related to the presence of PTA in TBI is important in clinical neuroscience. To date, however, these structures have not been clearly defined.

Diffusion tensor imaging (DTI) is a magnetic resonance imaging (MRI)-based technique that has an exceptional assessment advantage due to its ability to identify microstructural white matter abnormalities that are usually undetectable on conventional brain MRI^8–10^. DTI allows the evaluation of white matter tract integrity through its ability to image water diffusion characteristics^11–13^. Among the various DTI analytic methods, tract-based spatial statistics (TBSS) of fractional anisotropy (FA) values is an automated and sensitive technique that performs accurate voxel-based white matter analysis of multi-subject diffusion imaging data^14^. TBSS is a reliable and appropriate method that provides information on global changes in white matter microstructure^14^. However, there are no TBSS-based reports on the relationship between PTA duration and the white matter integrity in TBI.

In the current study, we investigated the relationship between the PTA duration and white matter integrity in TBI patients by undertaking TBSS.

## Methods

### Subjects

Forty-seven patients with TBI (22 men, 25 women; mean age 45.21 ± 17.68 years; range 20 ~ 79 years) and 47 age-and sex-matched normal control subjects (22 men, 25 women; mean age 40.60 ± 12.49 years; range 22 ~ 74 years) with no history of neurological/psychiatric disease were recruited to this study. The 47 patients with TBI were recruited according to the following inclusion criteria: (1) first-ever TBI, (2) age 20–79 years, (3) DTI scans obtained during the chronic stage (> 4 weeks after onset), and (4) no previous history neurologic/psychiatric disease. Those TBI patients with a continuous PTA state were excluded. Table 1 lists the demographic and clinical data of the patient and control groups. No significant difference in age or sex distribution was observed between the patient and control groups (*p* > 0.05). All procedures were performed following relevant guidelines and regulations. This study was performed retrospectively and conducted in accordance with the recommendations of the institutional review board of Yeungnam University Hospital. All of the patients and control subjects provided signed, informed consent and the institutional review board of Yeungnam University hospital approved the study protocol (ethical approval number: YUMC-2019-06-032).

**Table 1:** Demographic data for the patient and control groups.

	Patient group	Control group
Age (years)	45.21 ± 17.68	40.60 ± 12.49
Number (n)	47	47
Male:female	22:25	22:25
PTA duration (days)	8.05 ± 19.33	
LOC duration (days)	3.77 ± 10.67	
GCS score	12.66 ± 3.67	
MMSE score	25.28 ± 4.63	

*PTA* post-traumatic amnesia, *LOC* loss of consciousness, *GCS* Glasgow coma scale, *MMSE* mini-mental state examination; Values are means ± standard deviations.

### Cognitive function evaluation

A mini-mental state examination (MMSE) was performed to assess the subject’s cognitive function. MMSE evaluates orientation, memory, attention, calculation, visuospatial, and language abilities^15^. The maximum score is 30 points, and a score of 24 or lower indicates that a patient possibly has cognitive impairment^16^. The reliability and validity of the MMSE are well-established^15^. The MMSE score was obtained at the same time of DTI scanning (average of 7.43 ± 5.47 months after the onset of TBI).

### Diffusion tensor imaging

The DTI data were acquired at an average of 7.43 ± 5.47 months after head trauma onset using a 1.5 T Philips Gyroscan Intera (Hoffman-LaRoche, Best, Netherlands) with 32 non-collinear diffusion sensitizing gradients by performing single-shot echo-planar imaging. For each of the 32 non-collinear diffusion sensitizing gradients, 65 contiguous slices were acquired parallel to the anterior commissure–posterior commissure line^17^. The imaging parameters were as follows: acquisition matrix = 96 × 96, reconstructed to matrix = 192 × 192 matrix, field of view = 240 mm × 240 mm, TR = 10,398 ms, TE = 72 ms, parallel imaging reduction factor (SENSE factor) = 2, EPI factor = 59 and *b* = 1000 s/mm^2^, NEX = 1, slice gap = 0, and slice thickness = 2.5 mm^17^. Eddy current correction was performed to correct image distortion and head motion effects by using a diffusion registration package (Philips Medical Systems). Analysis of diffusion-weighted imaging data was performed by using tools within the Oxford Centre for Functional Magnetic Resonance Imaging of the Brain Software Library (www.fmrib.ox.ac.uk/fsl).

### Tract-based spatial statistics

Functional MRI assessment tools included in the FMRIB Software Library (FSL) were used to perform the data analyses. A previously described method was used to generate the fractional anisotropy (FA) maps^14^. Voxel-wise statistical analysis of the FA data was performed using TBSS, as implemented in FSL^18^. A nonlinear registration algorithm (www.doc.ic.ac.uk/~dr/software) was used to align the FA data for all subjects, obtained via FSL tools, to a template of average FA images (FMRIB-58) in Montreal Neurological Institute space. A mean FA image was produced and thinned to generate a mean FA skeleton representing the centers of all tracts common to the group members. A threshold was applied to a binarized mean FA skeleton at FA values > 0.2 before the resulting data were fed into the voxel-wise statistical analysis. The aligned FA data for each subject were then projected onto the mean skeleton, and voxel-wise cross-subject statistics were obtained to assess the differences between each group's FA values. The results were corrected for multiple comparisons by controlling for the family-wise error rate after performing threshold-free cluster enhancement. For further exploration of the data, the voxels identified by TBSS as showing substantial differences in each tract were selected, and the mean FA values for each subject were calculated. To summarize the FA data in a conventional neuroanatomical context and to identify relevant white matter tracts, mean FA values were calculated across the skeleton and within 48 regions of interest (ROIs), which were based on the intersections between the skeleton and the probabilistic Johns Hopkins University white matter atlases.

### Statistical analysis

Statistical analysis was performed using SPSS 21.0 for Windows (SPSS, Chicago, IL, USA). Multiple comparisons were corrected by the Benjamini–Hochberg (BH) procedure to control the false discovery rate, which represents the proportion of significant outcomes that are likely to be false positives^19^. To confirm the presence of white matter damage in the patient group, a multivariate analysis of variance (MANOVA) was performed to determine differences between the patient and control groups' FA values using Pillai’s trace that indicates a positive-valued statistic for contribution to the model^20^. The correlations between PTA duration and patient’s FA value, as well as between MMSE score and FA value, were evaluated by undertaking Pearson's correlation analysis; *p* < 0.05 and false discovery rate cut off < 0.1 were considered statistically significant.

## Results

**Figure 1:**
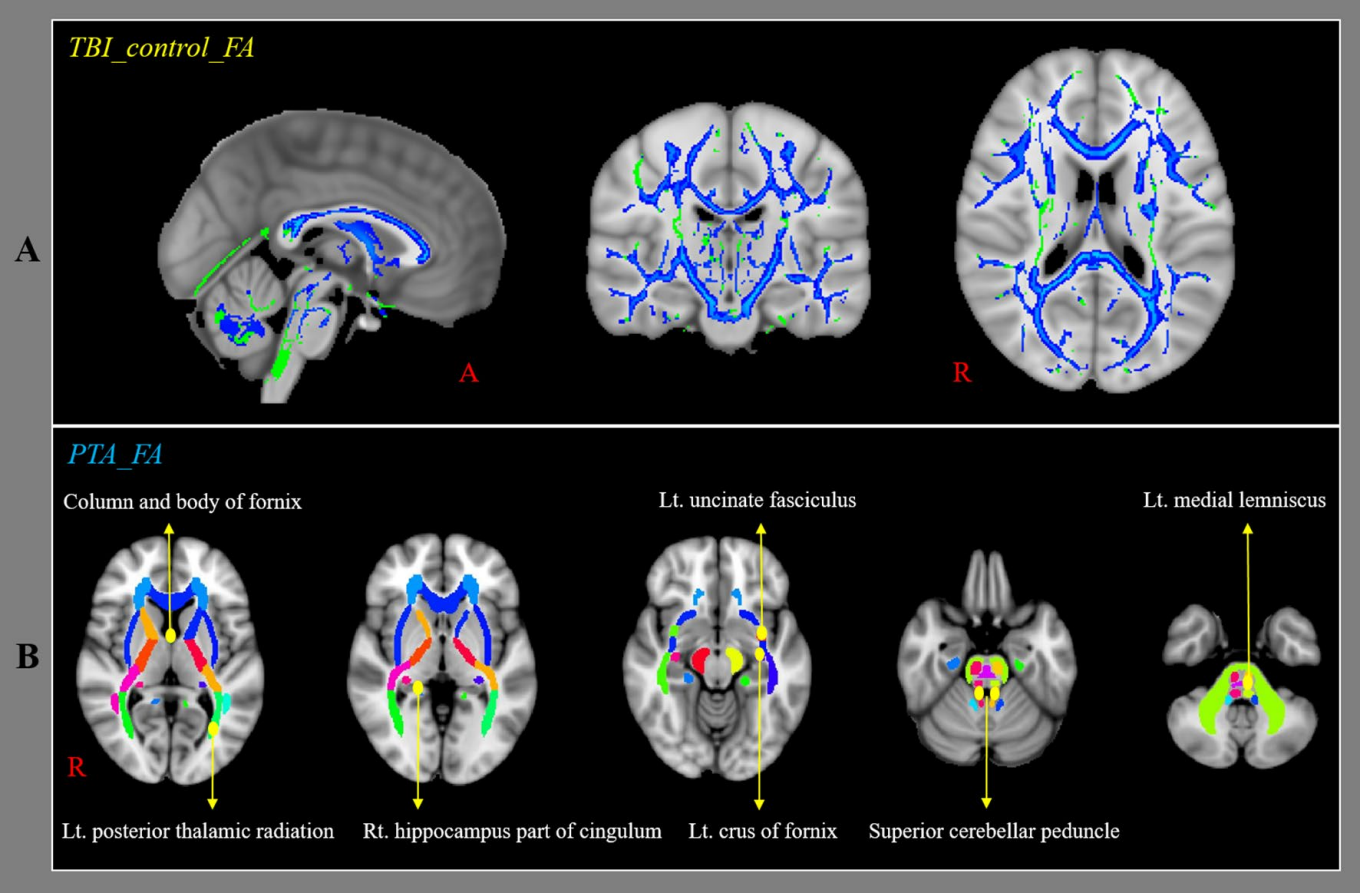
Results of tract-based spatial statistics analyses comparing the fraction anisotropy (FA) values of the patients and control groups and the correlation between post-traumatic amnesia (PTA) duration and FA values of patients. FA values were obtained for 48 regions of interest (ROIs) using a standard template of the John Hopkins University diffusion tensor imaging-based white matter atlases within the Functional Magnetic Resonance Imaging of the Brain Software Library (FSL version 5.1; www.fmrib.ox.ac.uk/fsl). (**a**) The blue voxels indicate areas with higher mean FA values in the control group than in the patient group. (**b**) Among the 48 ROIs, the FA values of eight ROIs (column and body of fornix, left crus of fornix, left uncinate fasciculus, right hippocampus part of cingulum, left medial lemniscus, right superior cerebellar peduncle, left superior cerebellar peduncle, left posterior thalamic radiation including optic radiation) in the patient group were negatively correlated with PTA.

Table 2 presents a comparison of the FA values of the patient and control groups. The FA values of 46 ROIs among the 48 ROIs showed significant differences between the patient and control groups (Pillai’s trace = 0.712, *p* < 0.05) (Fig. 1A). The BH correction revealed that the FA values of 46 ROIs among the 48 ROIs of the patient group were significantly different with those of the control group (BH *p* < 0.05). Table 3 lists the correlations between PTA duration and FA values and between MMSE score and FA values of the patients for the 48 ROIs. The FA values of eight of the 48 ROIs (column and body of fornix; *r* = − 0.345, left crus of fornix; *r* = − 0.315, left uncinate fasciculus; *r* = − 0.387, right hippocampus part of cingulum; *r* = − 0.429, left medial lemniscus; *r* = − 0.311, right superior cerebellar peduncle; *r* = − 0.290, left superior cerebellar peduncle; *r* = − 0.314, left posterior thalamic radiation (include optic radiation); *r* = − 0.323) in the patient group showed significant negative correlations to PTA duration (*p* < 0.05). There were no significant correlations between PTA duration and FA value in the remaining 40 ROIs of the patient group (*p* > 0.05) (Fig. 1B). The BH correction revealed that the FA values of two of the 48 ROIs (left uncinate fasciculus; *r* = − 0.387, right hippocampus part of cingulum; *r* = − 0.429) in the patient group showed significant negative correlations to PTA duration (BH *p* < 0.05). The FA values of nine of the 48 ROIs (body of corpus callosum; r = 0.312, splenium of corpus callosum; r = 0.296, column and body of fornix; r = 0.313, left crus of fornix; r = 0.304, left uncinate fasciculus; r = 0.300, right cingulate gyrus part of cingulum; r = 0.320, right hippocampus part of cingulum; r = 0.450, left posterior limb of internal capsule; r = 0.314, left tapetum; r = 0.326) in the patient group showed significant positive correlations to MMSE score without significant correlations in the remaining 39 ROIs of the patient group (*p* < 0.05). The BH correction revealed that the FA value of one of the 48 ROIs (right hippocampus part of cingulum; *r* = 0.450) in the patient group showed a significant positive correlation to MMSE score (BH *p* < 0.05). There was a significant negative correlation between PTA duration and MMSE score in the patient group (*r* = − 0.612; *p* < 0.05; BH *p* < 0.05).

**Table 2:** Comparison of the fractional anisotropy values of brain regions between the patient and control groups.

	Patient group	Control group	F	*p*-value	BH *p*-value
Genu of corpus callosum	0.49 ± 0.03	0.53 ± 0.02	43.73	0.000*	0.002**
Body of corpus callosum	0.52 ± 0.05	0.57 ± 0.03	31.44	0.000*	0.002**
Splenium of corpus callosum	0.64 ± 0.04	0.67 ± 0.02	29.38	0.000*	0.002**
Fornix (column and body)	0.32 ± 0.08	0.38 ± 0.07	15.97	0.000*	0.002**
Rt. fornix (crus)	0.40 ± 0.04	0.43 ± 0.03	13.97	0.000*	0.002**
Lt. fornix (crus)	0.43 ± 0.04	0.47 ± 0.03	19.89	0.000*	0.002**
Rt. uncinate fasciculus	0.41 ± 0.03	0.44 ± 0.03	19.80	0.000*	0.002**
Lt. uncinate fasciculus	0.40 ± 0.04	0.42 ± 0.03	20.60	0.000*	0.002**
Rt. superior longitudinal fasciculus	0.40 ± 0.02	0.42 ± 0.02	16.35	0.000*	0.002**
Lt. superior longitudinal fasciculus	0.40 ± 0.02	0.43 ± 0.02	27.01	0.000*	0.002**
Rt. superior fronto-occipital fasciculus	0.39 ± 0.05	0.43 ± 0.04	23.38	0.000*	0.002**
Lt. superior fronto-occipital fasciculus	0.37 ± 0.05	0.42 ± 0.04	26.02	0.000*	0.002**
Rt. cingulum (hippocampus)	0.33 ± 0.03	0.36 ± 0.03	16.23	0.000*	0.002**
Lt. cingulum (hippocampus)	0.34 ± 0.03	0.36 ± 0.03	10.37	0.002*	0.006**
Rt. cingulum (cingulate gyrus)	0.41 ± 0.03	0.44 ± 0.03	21.34	0.000*	0.002**
Lt. cingulum (cingulate gyrus)	0.44 ± 0.03	0.47 ± 0.02	23.50	0.000*	0.002**
Rt. anterior limb of internal capsule	0.47 ± 0.03	0.49 ± 0.02	34.59	0.000*	0.002**
Lt. anterior limb of internal capsule	0.46 ± 0.03	0.49 ± 0.02	40.96	0.000*	0.002**
Rt. posterior limb of internal capsule	0.56 ± 0.03	0.59 ± 0.02	20.35	0.000*	0.002**
Lt. posterior limb of internal capsule	0.57 ± 0.03	0.60 ± 0.02	32.32	0.000*	0.002**
Rt. retrolenticular part of internal capsule	0.49 ± 0.03	0.51 ± 0.03	9.59	0.003*	0.008**
Lt. retrolenticular part of internal capsule	0.50 ± 0.03	0.52 ± 0.02	12.31	0.001*	0.004**
Rt. external capsule	0.34 ± 0.02	0.36 ± 0.02	24.23	0.000*	0.002**
Lt. external capsule	0.35 ± 0.02	0.38 ± 0.02	28.77	0.000*	0.002**
Rt. Inferior cerebellar peduncle	0.41 ± 0.02	0.42 ± 0.03	3.86	0.053	0.015
Lt. Inferior cerebellar peduncle	0.40 ± 0.03	0.41 ± 0.03	3.82	0.054	0.017
Rt. superior cerebellar peduncle	0.48 ± 0.04	0.52 ± 0.02	25.52	0.000*	0.002**
Lt. superior cerebellar peduncle	0.46 ± 0.04	0.50 ± 0.02	28.56	0.000*	0.002**
Middle cerebellar peduncle	0.43 ± 0.02	0.46 ± 0.02	59.95	0.000*	0.002**
Rt. posterior thalamic radiation (include optic radiation)	0.50 ± 0.04	0.53 ± 0.03	12.25	0.001*	0.004**
Lt. posterior thalamic radiation (include optic radiation)	0.50 ± 0.04	0.53 ± 0.03	13.65	0.000*	0.002**
Rt. medial lemniscus	0.53 ± 0.03	0.55 ± 0.03	7.28	0.008*	0.010**
Lt. medial lemniscus	0.53 ± 0.03	0.55 ± 0.03	7.12	0.009*	0.013**
Rt. tapetum	0.33 ± 0.04	0.47 ± 0.03	18.61	0.000*	0.002**
Lt. tapetum	0.26 ± 0.02	0.28 ± 0.02	20.47	0.000*	0.002**
Pontine crossing tract	0.41 ± 0.03	0.43 ± 0.02	20.06	0.000*	0.002**
Rt. corticospinal tract	0.46 ± 0.03	0.51 ± 0.03	44.50	0.000*	0.002**
Lt. corticospinal tract	0.48 ± 0.05	0.53 ± 0.03	34.00	0.000*	0.002**
Rt. cerebral peduncle	0.58 ± 0.03	0.61 ± 0.03	33.58	0.000*	0.002**
Lt. cerebral peduncle	0.58 ± 0.04	0.62 ± 0.02	31.18	0.000*	0.002**
Rt. anterior corona radiata	0.37 ± 0.03	0.40 ± 0.03	17.90	0.000*	0.002**
Lt. anterior corona radiata	0.37 ± 0.04	0.41 ± 0.03	25.98	0.000*	0.002**
Rt. superior corona radiata	0.41 ± 0.03	0.43 ± 0.03	18.96	0.000*	0.002**
Lt. superior corona radiata	0.41 ± 0.03	0.44 ± 0.02	20.29	0.000*	0.002**
Rt. posterior corona radiata	0.41 ± 0.03	0.44 ± 0.02	27.15	0.000*	0.002**
Lt. posterior corona radiata	0.40 ± 0.03	0.43 ± 0.02	28.93	0.000*	0.002**
Rt. sagittal stratum	0.45 ± 0.03	0.48 ± 0.02	17.31	0.000*	0.002**
Lt. sagittal stratum	0.43 ± 0.03	0.45 ± 0.02	15.43	0.000*	0.002**

*Significant differences between the patient and control groups; patient group < control group, *p* < 0.05.

**Significant differences between the patient and control groups using a Benjamini–Hochberg (BH) correction; Values are means ± standard deviations.

**Table 3:** Correlation between the post-traumatic amnesia duration and fractional anisotropy values and between mini-mental state examination score and fractional anisotropy values of the patient group.

	PTA–FA			MMSE–FA		
	*r*-value	*p*-value	BH *p*-value	*r*-value	*p*-value	BH *p*-value
Genu of corpus callosum	− 0.183	0.219	0.108	0.282	0.055	0.032
Body of corpus callosum	− 0.230	0.119	0.078	0.312	0.033*	0.022
Splenium of corpus callosum	− 0.232	0.117	0.076	0.296	0.043*	0.028
Fornix (column and body)	− 0.345	0.018*	0.010	0.313	0.032*	0.020
Rt. fornix (crus)	− 0.259	0.078	0.048	0.253	0.086	0.054
Lt. fornix (crus)	− 0.315	0.031*	0.018	0.304	0.038*	0.024
Rt. uncinate fasciculus	− 0.265	0.072	0.042	0.241	0.102	0.062
Lt. uncinate fasciculus	− .0.387	0.007*	0.008**	0.300	0.040*	0.026
Rt. superior longitudinal fasciculus	− 0.051	0.731	0.178	0.180	0.227	0.114
Lt. superior longitudinal fasciculus	− 0.057	0.702	0.166	0.094	0.531	0.158
Rt. superior fronto-occipital fasciculus	− 0.157	0.292	0.130	0.147	0.325	0.134
Lt. superior fronto-occipital fasciculus	− 0.141	0.343	0.140	0.054	0.720	0.170
Rt. cingulum (hippocampus)	− 0.429	0.003*	0.006**	0.450	0.002*	0.004**
Lt. cingulum (hippocampus)	− 0.172	0.249	0.154	0.131	0.382	0.088
Rt. Cingulum (cingulate gyrus)	− 0.138	0.357	0.122	0.320	0.028*	0.148
Lt. cingulum (cingulate gyrus)	− 0.175	0.240	0.144	0.239	0.105	0.016
Rt. anterior limb of internal capsule	− 0.141	0.344	0.118	0.085	0.571	0.068
Lt. anterior limb of internal capsule	− 0.174	0.242	0.142	0.144	0.334	0.160
Rt. posterior limb of internal capsule	− 0.224	0.129	0.120	0.241	0.103	0.138
Lt. posterior limb of internal capsule	− 0.274	0.062	0.082	0.314	0.032*	0.064
Rt. retrolenticular part of internal capsule	0.053	0.725	0.128	0.055	0.715	0.058
Lt. retrolenticular part of internal capsule	− 0.131	0.379	0.098	0.180	0.226	0.092
Rt. external capsule	− 0.187	0.208	0.034	0.221	0.135	0.020
Lt. external capsule	− 0.197	0.186	0.172	0.186	0.210	0.168
Rt. Inferior cerebellar peduncle	− 0.080	0.592	0.174	0.207	0.162	0.176
Lt. Inferior cerebellar peduncle	− 0.149	0.318	0.050	0.238	0.107	0.100
Rt. superior cerebellar peduncle	− 0.290	0.048*	0.146	0.209	0.158	0.112
Lt. superior cerebellar peduncle	− 0.314	0.032*	0.104	0.181	0.224	0.084
Middle cerebellar peduncle	− 0.231	0.119	0.102	0.227	0.125	0.106
Rt. Posterior thalamic radiation (include optic radiation)	− 0.251	0.089	0.048	0.144	0.333	0.046
Lt. posterior thalamic radiation (include optic radiation)	− 0.323	0.027*	0.162	0.233	0.115	0.094
Rt. medial lemniscus	− 0.269	0.068	0.082	0.247	0.095	0.096
Lt. medial lemniscus	− 0.311	0.033*	0.086	0.271	0.065	0.116
Rt. tapetum	− 0.262	0.075	0.132	0.235	0.111	0.070
Lt. tapetum	− 0.165	0.268	0.030	0.326	0.026*	0.090
Pontine crossing tract	− 0.095	0.526	0.020	0.074	0.620	0.110
Rt. corticospinal tract	− 0.109	0.468	0.078	0.108	0.472	0.080
Lt. corticospinal tract	− 0.167	0.262	0.056	− 0.001	0.993	0.136
Rt. cerebral peduncle	− 0.256	0.082	0.014	0.266	0.071	0.074
Lt. cerebral peduncle	− 0.253	0.086	0.038	0.240	0.104	0.060
Rt. Anterior corona radiata	− 0.199	0.180	0.022	0.209	0.159	0.036
Lt. anterior corona radiata	− 0.259	0.078	0.044	0.262	0.076	0.072
Rt. superior corona radiata	− 0.097	0.517	0.126	0.211	0.154	0.012
Lt. superior corona radiata	− 0.160	0.282	0.156	0.248	0.093	0.164
Rt. posterior corona radiata	− 0.225	0.129	0.150	0.206	0.165	0.152
Lt. posterior corona radiata	− 0.219	0.139	0.124	0.176	0.237	0.180
Rt. sagittal stratum	− 0.052	0.728	0.052	0.052	0.730	0.040
Lt. sagittal stratum	− 0.258	0.079	0.054	0.198	0.183	0.066

*PTA* post-traumatic amnesia, *MMSE* mini-mental state examination, *FA* fractional anisotropy.

*Significant correlation between the post-traumatic amnesia duration and fractional anisotropy values, and the mini-mental state examination and fractional anisotropy values, *p* < 0.05.

**Significant correlation between the post-traumatic amnesia duration and fractional anisotropy values, and the mini-mental state examination and fractional anisotropy values using a Benjamini–Hochberg (BH) correction; Values are means ± standard deviations.

## Discussion

This study examined the relationship between PTA duration and white matter integrity in TBI patients by using TBSS-based analysis. The results are summarized as follows. First, both before and after the BH corrections, the FA values of 46 ROIs, among the 48 ROIs assessed, were lower in the patient group than in the control group. Second, the PTA duration was negatively correlated with the FA values of the column, body and left crus of fornix, left uncinate fasciculus, right hippocampus part of cingulum, right superior cerebellar peduncle, left superior cerebellar peduncle, left posterior thalamic radiation (include optic radiation), and left medial lemniscus regions. After the BH correction, the PTA duration was negatively correlated with the FA values of the left uncinate fasciculus and right hippocampus part of cingulum regions. Third, in the patient group, the MMSE score was positively correlated with the FA values of the body and splenium of corpus callosum, column, body and left crus of fornix, left uncinate fasciculus, right cingulate part and right hippocampus part of cingulum, left posterior limb of internal capsule, and left tapetum regions. After the BH correction, the MMSE score was positively correlated with the FA value of the right hippocampus part of cingulum region. Fourth, both before and after the BH corrections, PTA duration was negatively correlated with MMSE score in the patient group.

The FA value represents the degree of directionality of water diffusion and indicates the integrity of white matter microstructures, such as axons, myelin, and microtubules^21^. Thus, a decrease in the FA value of a neural structure represents a decrease in the neural structure's microstructural integrity^11–13^. Therefore, the study results showing lower FA values in 46 of the 48 ROIs assessed in the patient group both before and after the BH corrections indicate there is lower microstructural integrity in these neural structures, suggesting the presence of neural injury.

Regarding the correlation between the PTA duration and FA values in the patient group, the FA values of eight white matter neural structures that are related to cognitive function were negatively correlated with PTA duration^22–30^. The fornix is involved in episodic memory recall, and the uncinate fasciculus functions are related to learning and memory^22–24^. The cingulum has been reported to be an important neural structure for episodic memory and executive control^25^, and the superior cerebellar peduncle is involved in the social cognitive neural network, acting as a major pathway for cerebro-cerebellar connections. In contrast, the posterior thalamic radiation, including the optic radiation, is involved in visuomotor task performance^26,29^. Regarding the medial lemniscus, lesions at the thalamus and prefrontal levels can cause executive dysfunction because this area is involved in a pathway that reaches the prefrontal cortex via the thalamus^27,28,30^. The PTA–FA correlation results suggest that PTA duration in TBI is closely related to the injury severity of the above eight neural structures that are involved in cognitive function. In addition, the results showing positive correlations between MMSE score and FA values of the fornix, uncinate fasciculus, and cingulum in the patient group indicate relationships between cognitive ability and FA values in those neural structures. The revelation of a significant correlation between PTA duration and MMSE score in the patient group both before and after the BH corrections further indicates that PTA duration is associated with cognitive function, and that correlation is consistent with the correlation between PTA duration and FA value of the neural structures involved in cognitive function. Furthermore, after the BH correction, the results that the uncinate fasciculus and hippocampus part of cingulum were negatively correlated with PTA duration, and the hippocampus part of cingulum was positively correlated with MMSE score, demonstrated that the uncinate fasciculus and hippocampus part of cingulum were more closely related to PTA duration and cognitive ability. However, the white matter neural structures that did not negatively correlated with PTA duration after the BH correction appeared to be lack of the sensitivity necessary to detect relationship with PTA duration^31^.

Many studies have described relationships between PTA duration and neuropsychological outcomes in TBI^32–41^. These studies have focused on the association between PTA duration and cognitive outcomes, such as verbal, intelligence, learning and memory, speed of information processing, and perceptual or constructional skills. On the other hand, many DTI studies have reported a relationship between cognitive performance and white matter integrity in patients with TBI by examining TBSS results^42–48^. These studies have reported relationships between white matter integrity and various cognitive functions, such as memory, associative learning, execution, and social function. However, the present study is the first to demonstrate a relationship between PTA duration and subsequent white matter integrity in patients with TBI. Nevertheless, some limitations of this study should be considered. First, various evaluation methods can assess cognitive ability, but this study, because it was retrospectively conducted, only used the MMSE to indicate cognitive function. Second, although DTI data were obtained during the chronic stage, duration from onset to DTI scanning was heterogeneous. Further prospective studies using detailed neuropsychological testing and considering more homogeneous duration from onset to DTI scanning should be encouraged. Third, the TBSS approach reduces white matter tracts to a skeleton framework, thereby delineating the center of the tract and projecting onto that center only the highest value FA along that projection, resulting in a loss of information and the potential presence of artifacts^49,50^. Fourth, TBSS-based methods calculate statistics only for skeleton voxels, resulting in few statistical comparisons in less-relevant voxels. Therefore, the effects of family-wise error correction are reduced^50^.

In conclusion, this study examined the relationship between PTA duration and white matter integrity in TBI patients by undertaking TBSS-based analysis. In patients with TBI, PTA duration was related to injury severity in eight neural structures (especially, the uncinate fasciculus and hippocampus part of cingulum) involved in cognitive function. The results suggest that PTA duration can indicate injury severity of the above neural structures in TBI patients.

## References

[1] Russell WR, Smith A (1961). Post-traumatic amnesia in closed head injury. Arch Neurol..

[2] Tate RL (2006). A multicentre, randomised trial examining the effect of test procedures measuring emergence from post-traumatic amnesia. J. Neurol. Neurosurg. Psychiatry..

[3] Group TCmW. VA/DoD clinical practice guideline for management of concussion/mild traumatic brain injury. *J. Rehabil. Res. Dev*. **46**, CP-168 (2009). 20108447

[4] Marshman LA, Jakabek D, Hennessy M, Quirk F, Guazzo EP (2013). Post-traumatic amnesia. J. Clin. Neurosci..

[5] Brown AW (2005). Clinical elements that predict outcome after traumatic brain injury: A prospective multicenter recursive partitioning (decision-tree) analysis. J. Neurotrauma..

[6] Sherer M, Struchen MA, Yablon SA, Wang Y, Nick TG (2008). Comparison of indices of traumatic brain injury severity: Glasgow coma scale, length of coma and post-traumatic amnesia. J. Neurol. Neurosurg. Psychiatry..

[7] Walker WC (2010). A multicentre study on the clinical utility of post-traumatic amnesia duration in predicting global outcome after moderate-severe traumatic brain injury. J. Neurol. Neurosurg. Psychiatry..

[8] Liu YW (2007). Subarachnoid hemorrhage in the subacute stage: Elevated apparent diffusion coefficient in normal-appearing brain tissue after treatment. Radiology.

[9] Jang SH, Kim SH, Lim HW, Yeo SS (2014). Injury of the lower ascending reticular activating system in patients with hypoxic-ischemic brain injury: Diffusion tensor imaging study. Neuroradiology.

[10] O'Phelan KH, Otoshi CK, Ernst T, Chang LD (2018). Common patterns of regional brain injury detectable by diffusion tensor imaging in otherwise normal-appearing white matter in patients with early moderate to severe traumatic brain injury. J. Neurotrauma..

[11] Basser PJ, Mattiello J, LeBihan D (1994). Estimation of the effective self-diffusion tensor from the NMR spin echo. J. Magn. Reson. B..

[12] Mori S, Crain BJ, Chacko VP, van Zijl PC (1999). Three-dimensional tracking of axonal projections in the brain by magnetic resonance imaging. Ann. Neurol..

[13] Alexander AL, Lee JE, Lazar M, Field AS (2007). Diffusion tensor imaging of the brain. Neurotherapeutics.

[14] Smith SM (2006). Tract-based spatial statistics: Voxelwise analysis of multi-subject diffusion data. Neuroimage.

[15] Folstein MF, Folstein SE, McHugh PR (1975). "Mini-mental state". A practical method for grading the cognitive state of patients for the clinician. J. Psychiatr. Res..

[16] Matsuura T (2019). Statistical analysis of dual-task gait characteristics for cognitive score estimation. Sci. Rep..

[17] Lee SJ, Kim MS, Jang SH (2020). White matter abnormalities in spontaneous subarachnoid hemorrhage: A tract-based spatial statistics study. Stroke.

[18] Anjari M (2007). Diffusion tensor imaging with tract-based spatial statistics reveals local white matter abnormalities in preterm infants. Neuroimage.

[19] Benjamini Y, Hochberg Y (1995). Controlling the false discovery rate: A practical and powerful approach to multiple testing. J. R. Stat. Soc. Ser. B..

[20] Olson CL (1974). Comparative robustness of six tests in multivariate analysis of variance. J. Am. Stat. Assoc..

[21] Assaf Y, Pasternak O (2008). Diffusion tensor imaging (DTI)-based white matter mapping in brain research: A review. J. Mol. Neurosci..

[22] Van der Werf YD (2003). Deficits of memory, executive functioning and attention following infarction in the thalamus; A study of 22 cases with localised lesions. Neuropsychologia.

[23] Funahashi S, Andreau JM (2013). Prefrontal cortex and neural mechanisms of executive function. J. Physiol. Paris..

[24] Alm KH, Rolheiser T, Mohamed FB, Olson IR (2015). Fronto-temporal white matter connectivity predicts reversal learning errors. Front. Hum. Neurosci..

[25] Metoki A, Alm KH, Wang Y, Ngo CT, Olson IR (2017). Never forget a name: White matter connectivity predicts person memory. Brain Struct. Funct..

[26] Bubb EJ, Metzler-Baddeley C, Aggleton JP (2018). The cingulum bundle: Anatomy, function, and dysfunction. Neurosci. Biobehav. Rev..

[27] Ryan NP (2018). White matter microstructure predicts longitudinal social cognitive outcomes after paediatric traumatic brain injury: A diffusion tensor imaging study. Psychol. Med..

[28] Subramaniam K (2018). White matter microstructure predicts cognitive training-induced improvements in attention and executive functioning in schizophrenia. Schizophr. Res..

[29] Yin B (2019). Longitudinal changes in diffusion tensor imaging following mild traumatic brain injury and correlation with outcome. Front. Neural Circuits..

[30] Senova S, Fomenko A, Gondard E, Lozano AM (2020). Anatomy and function of the fornix in the context of its potential as a therapeutic target. J. Neurol. Neurosurg. Psychiatry..

[31] Bigler ED, Brooks M (2009). Traumatic brain injury and forensic neuropsychology. J. Head Trauma Rehabil..

[32] Mandleberg IA (1975). Cognitive recovery after severe head injury. 2. Wechsler adult intelligence scale during post-traumatic amnesia. J. Neurol. Neurosurg. Psychiatry..

[33] Mandleberg IA (1976). Cognitive recovery after severe head injury. 3. WAIS verbal and performance IQs as a function of post-traumatic amnesia duration and time from injury. J. Neurol. Neurosurg. Psychiatry..

[34] Brooks DN, Aughton ME, Bond MR, Jones P, Rizvi S (1980). Cognitive sequelae in relationship to early indices of severity of brain damage after severe blunt head injury. J. Neurol. Neurosurg. Psychiatry..

[35] Shores EA (1989). Comparison of the westmead PTA Scale and the glasgow coma scale as predictors of neuropsychological outcome following extremely severe blunt head injury. J. Neurol. Neurosurg. Psychiatry..

[36] Geffen GM, Encel JS, Forrester GM (1991). Stages of recovery during post-traumatic amnesia and subsequent everyday memory deficits. NeuroReport.

[37] Haslam C (1994). Post-coma disturbance and post-traumatic amnesia as nonlinear predictors of cognitive outcome following severe closed head injury: Findings from the westmead head injury project. Brain Inj..

[38] Haslam C, Batchelor J, Fearnside MR, Haslam SA, Hawkins S (1995). Further examination of post-traumatic amnesia and post-coma disturbance as nonlinear predictors of outcome after head-injury. Neuropsychology.

[39] McFarland K, Jackson L, Geffe G (2001). Post-traumatic amnesia: Consistency-of-recovery and duration-to-recovery following traumatic brain impairment. Clin. Neuropsychol..

[40] Formisano R (2004). Clinical predictors and neuropsychological outcome in severe traumatic brain injury patients. Acta Neurochir. (Wien)..

[41] Sigurdardottir S (2015). Neuropsychological functioning in a national cohort of severe traumatic brain Injury: Demographic and acute injury-related predictors. J. Head Trauma Rehabil..

[42] Kinnunen KM (2011). White matter damage and cognitive impairment after traumatic brain injury. Brain.

[43] Spitz G, Maller JJ, O'Sullivan R, Ponsford JL (2013). White matter integrity following traumatic brain injury: The association with severity of injury and cognitive functioning. Brain Topogr..

[44] Xiong KL (2014). White matter integrity and cognition in mild traumatic brain injury following motor vehicle accident. Brain Res..

[45] Owens JA, Spitz G, Ponsford JL, Dymowski AR, Willmott C (2018). An investigation of white matter integrity and attention deficits following traumatic brain injury. Brain Inj..

[46] Kim E (2019). Altered white matter integrity after mild to moderate traumatic brain injury. J. Clin. Med..

[47] McDonald S, Dalton KI, Rushby JA, Landin-Romero R (2019). Loss of white matter connections after severe traumatic brain injury (TBI) and its relationship to social cognition. Brain Imaging Behav..

[48] Yiannakkaras C (2019). Whole brain and corpus callosum diffusion tensor metrics: How do they correlate with visual and verbal memory performance in chronic traumatic brain injury. J. Integr. Neurosci..

[49] Zalesky A (2011). Moderating registration misalignment in voxelwise comparisons of DTI data: A performance evaluation of skeleton projection. Magn. Reson. Imaging..

[50] Schwarz CG (2014). Improved DTI registration allows voxel-based analysis that outperforms Tract-Based Spatial Statistics. Neuroimage.

